# A Priori Knowledge and Probability Density Based Segmentation Method for Medical CT Image Sequences

**DOI:** 10.1155/2014/769751

**Published:** 2014-05-19

**Authors:** Huiyan Jiang, Hanqing Tan, Benqiang Yang

**Affiliations:** ^1^Software College, Northeastern University, Shenyang 110819, China; ^2^Radiology Department, PLA General Hospital, Shenyang 110016, China

## Abstract

This paper briefly introduces a novel segmentation strategy for CT images sequences. As first step of our strategy, we extract a priori intensity statistical information from object region which is manually segmented by radiologists. Then we define a search scope for object and calculate probability density for each pixel in the scope using a voting mechanism. Moreover, we generate an optimal initial level set contour based on a priori shape of object of previous slice. Finally the modified distance regularity level set method utilizes boundaries feature and probability density to conform final object. The main contributions of this paper are as follows: a priori knowledge is effectively used to guide the determination of objects and a modified distance regularization level set method can accurately extract actual contour of object in a short time. The proposed method is compared to other seven state-of-the-art medical image segmentation methods on abdominal CT image sequences datasets. The evaluated results demonstrate our method performs better and has the potential for segmentation in CT image sequences.

## 1. Introduction

Organ segmentation is a crucial step prior to computer-aided diagnosis, since it is fundamental for further medical image processing such as cancer detection, lesion recognition, and three-dimensional visualization. However, organ extraction is considered as a challenge task due to huge shape variations, heterogeneous intensity distribution, and low contrast of CT image [1]. Especially complicated surrounding and weak edge cause serious impediment to accurately segment pancreas.

Various methods are proposed to solve the medical image segmentation problem. The main categories of these methods can be classified as statistical shape model (SSM) [2], level set [3–8], probabilistic atlases [9], histogram-based approaches [10], and region growing method [11, 12].

The statistical shape model and probabilistic atlases seriously depend on the shape and intensity distribution of objects in training dataset, so that they suffer from large variations of shape and intensity. The histogram-based approaches always use a classification system to differentiate target object from other tissues; the leakage problem exists in these systems.

Level set methods can represent complex topology of contours and handle topological changes in a natural and effective way, such that various level set methods are proposed to solve the medical image segmentation problem. The shape detection level set method [3] applies a shape modeling scheme in level set evolution. The geodesic active contour (GAC) [4] model employs edge feature to guide segmentation. However these edge-based level set methods easily cause leakage in weak boundaries of objects. The C-V model [5] which seeks global optimization is not suitable for local optimization segmentation. A hybrid level set method [6] combines both boundary and region information to achieve segmentation results. It utilizes a predefined parameter to indicate the lower bound of the gray level of the target object in region term. Its boundary term is similar to the one in GAC method. However its predefined parameter is not easy to be accurately defined and reinitialization of zero level set is needed. A priori shape based level set method [7] uses a priori shape knowledge to guide the segmentation, but it suffers from large variations of shape and intensity distribution. Moreover, level set methods have a high requirement to locate initial zero level set near final contour. The similarity between nearby slices in CT image sequences is ignored in level set methods. The problem of leakage easily happens in weak boundary area.

In order to solve these problems, this paper proposes a novel segmentation strategy that regards similarity of intensity distribution, shape, and location between nearby slices as a priori knowledge to guide the segmentation of image sequences. The kernel of this paper is that a probability density map which is generated using the novel application strategy of a priori knowledge is used to modify a distance regularization level set method. The proposed method is compared to geodesic active contour model, C-V model, shape detection level set method, the hybrid level set method, and confident connected region growing method. Finally the novel method is compared to our previous improved variational level set method [8]. The evaluated results prove that our method is effective to segment organs from abdominal CT image sequences. The rest of this paper is arranged as follows. The proposed method is explained in Section 2. Evaluation and discussion of our method are presented in Section 3, and Section 4 concludes this paper.

## 2. Materials and Methods

### 2.1. Distance Regularity Level Set

A distance regularity level set method is proposed in [13]. This method inherently maintains a signed distance profile near the zero level set, such that it eliminates the requirement of reinitialization of level set function. It is able to provide accurate numerical calculation in level set evolution.

The energy function of level set is define by

(1) $$\begin{array}{c} E\left(\phi \right)=\mu R_{p}\left(\phi \right)+\beta \eta \left(\phi \right), \end{array}$$

where *μ* > 0 is a constant, *R*_*p*_(*ϕ*) is level set distance regularization term, and *η*(*ϕ*) is external force term.

*R*_*p*_(*ϕ*) is defined in [11] by

(2) $$\begin{array}{c} R_{p}\left(\phi \right)≜\int_{\Omega }p\left(\left|\nabla \phi \right|\right)dx, \end{array}$$

where *p* is a double-well potential function for the distance regularization term *R*_*p*_ and is constructed as

(3) $$\begin{array}{c} p\left(s\right)=\{\begin{array}{cc} \frac{1}{{\left(2\pi \right)}^{2}}\left(1-\cos\left(2\pi s\right)\right), & \text{if}\;\;s\leq 1\\ \frac{1}{2}{\left(s-1\right)}^{2}, & \text{if}\;\;s>1. \end{array} \end{array}$$

*δ*_*ε*_ and *H*_*ε*_ are smooth functions in level set methods proposed in [14, 15]. Moreover, *H*_*ε*_′ = *δ*_*ε*_ and *ε* is set to 1.5.

(4) $$\begin{array}{c} \delta_{\varepsilon }\left(x\right)=\{\begin{array}{cc} \frac{1}{2\varepsilon }\left[1+\cos\left(\frac{\pi x}{\varepsilon }\right)\right] & \left|x\right|\leq \varepsilon \\ 0 & \left|x\right|>\varepsilon , \end{array}\\ H_{\varepsilon }\left(x\right)=\{\begin{array}{cc} \frac{1}{2}\left(1+\frac{x}{\varepsilon }+\frac{1}{\pi }\sin\left(\frac{\pi x}{\varepsilon }\right)\right) & \left|x\right|\leq \varepsilon \\ 1 & x>\varepsilon \\ 0 & x<-\varepsilon . \end{array} \end{array}$$

The *R*_*p*_(*ϕ*) makes the level set evolution have a unique forward-and-backward diffusion effect, which eliminates the need for reinitialization, such that its induced numerical errors are avoided. Therefore level set evolution is more stable and robust.

### 2.2. A Priori Information Extraction

The traditional a priori knowledge such as shape and intensity distribution is always extracted from training dataset, which represents the commonness of object but cannot directly represent the individual characteristics of the current object in medical image. The differences between commonness and individuality usually cause errors in finial segmentation results. Moreover, the large variation of shape and intensity distribution of organs bring a great difficulties in using traditional commonness to guide the segmentation.

In order to overcome these problems, a new scheme is proposed to extract the individuality feature of object as a priori knowledge which is then employed to optimize the segmentation process of level set method. As the first step of processing, we check through the input abdominal CT volume to find out a slice in which object organs have a largest cross-section. A radiologist defines the boundary of organs in this slice. The shape of boundary and the intensity distribution parameters of this object organ region are used as a priori knowledge in the next step of segmentation.

Though variation of shape and intensity is obvious between different volumes or slices that have a large imaging distance in the same volume, these features in neighbor slices which belong to the same volume are similar. Thus, we follow the a priori shape of previous slice to segment next slice. The statistics dataset is initial as the manually segmented slice. Subsequent segmented results will be added into the statistics dataset as statistical sample.

Each segmented sample in the training dataset is regarded as a scope of statistics. Mean intensity and intensity variance for each sample are calculated:

(5) $$\begin{array}{c} u=\frac{1}{n}\overset{n}{\underset{i=1}{\sum }}p_{i}\;p_{i}\in R_{s},\\ \sigma =\frac{1}{n}\sqrt{\overset{n}{\underset{i=1}{\sum }}{\left(p_{i}-u\right)}^{2}}\;p_{i}\in R_{s}. \end{array}$$

*p*_*i*_ is the intensity value of pixels in samples. All the pairs of parameters make a statistical feature set *F* = {(*u*_*i*_, *σ*_*i*_) | *i* = 1,2,…, *n*}, which plays an important role in processing of segmentation. Through amount of statistical experiments, the statistics indicate that about 92.4% of pixels in object region is located in [*μ* − 2*σ*, *μ* + 2*σ*].

### 2.3. A Priori Based Distance Regularity Level Set

Some defects exist in the original distance regularization level set method. It is sensitive to initial position of the zero level set contour. The initial zero level set is required to locate near the final contour. Otherwise, the curve evolution needs amount of iterative calculation to pull curve toward object contour. Moreover, original distance regularization level set method has oversegmentation problem of leakages into nearby tissue in weak boundary area. Especially object is always connected to neighbor organs and boundary usually is fuzzy in CT image; the original method cannot get satisfactory results in most case.

In order to solve these problems, we employ a priori statistical feature to modify distance regularity level set as well as confirming an optimal initial level set. Then the modified method is used to extract the object organ from CT images.

The statistical information which comes from statistical dataset is added into the external energy term of energy function of level set, such that new energy function is defined as

(6) $$\begin{array}{c} E\left(\phi \right)=\mu R_{p}\left(\phi \right)+\alpha S\left(\phi \right)+\lambda L_{g}\left(\phi \right), \end{array}$$

where the first term is distance regularization term, the second and third terms are external energy terms, which are used to pull the initial curve toward the final object curve in evolution. *λ* > 0 and *α* ∈ *R* are coefficients to control the weight of external energy. *L*_*g*_(*ϕ*) depends on image gradient information and *S*(*ϕ*) relays on a priori statistical feature. They correspond to *η*(*ϕ*) in function (1).

**Figure 1:**
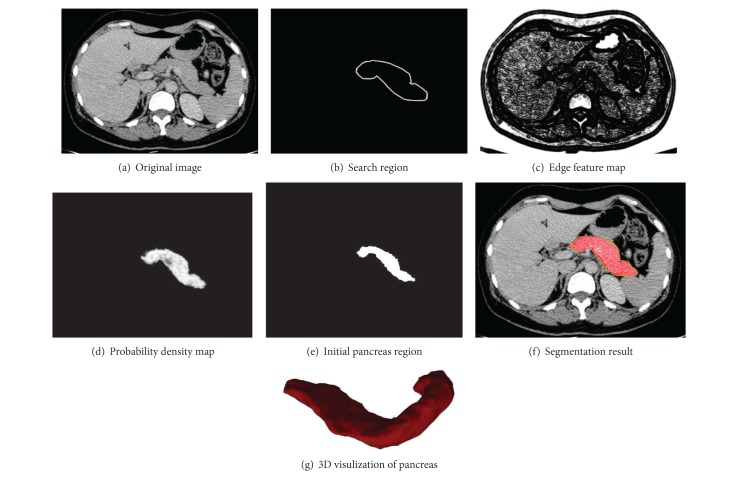
Segmentation example: necessary reprocessing for pancreas segmentation. (a) Denoised CT image. (b) The mask of previous slice. (c) The search region of pancreas based on mask of previous slice. (d) Edge feature map. (e) Probability density map. (f) Initial pancreas region used in our level set method.

*S*(*ϕ*) is defined as

(7) $$\begin{array}{c} S\left(\phi \right)=\int_{\Omega }s\left(I_{m}\right)H\left(-\phi \right)dx\;dy, \end{array}$$

where *I*_*m*_ = *MI* is a search area which contains all pixels of current object region. *M* is a mask function used to define a search domain which includes object organ in the CT slice *I*. The mask *M* derives from the extracted object region of previous slice of current slice *I*. The previous object region extends outward *n* pixel along its shape to generate the mask scope (see Figure 1). The pixels inside the scope are set to 1 and those outside the scope set to 0. Since the location and shape are similar between two contiguous slices, *s*(*I*_*m*_) is a similarity measure function. It estimates the probability of belonging to object tissue of each pixel in search area.

In order to measure the similarity, first a probability density formula is defined as

(8) $$\begin{array}{c} p\left(x\right)=\{\begin{array}{cc} e^{-{\left(x-\mu \right)}^{2}/2\sigma^{2}}, & x\in \left[\mu -2\sigma ,\mu +2\sigma \right]\\ -\frac{\left|x-\mu \right|}{2\sigma }, & \text{otherwise}, \end{array} \end{array}$$

where *p*(*x*) is probability density. *x* is an intensity value of pixel within search area. *μ* is mean intensity, and *σ* is intensity variance. They come from statistical feature set *F*. For each pixel within search area, a set of probability density *P* = {*p*_1_, *p*_2_,…, *p*_*n*_} is calculated based on all statistical features {(*u*_*i*_, *σ*_*i*_) | *i* = 1,2,…, *n*}.

A voting mechanism is employed to determine the actual probability density of a pixel. The voting mechanism is defined as

(9) $$\begin{array}{c} \text{Votes}\left(F\right)≜\{\begin{array}{cc} V_{x,a}+1 & x\in \left[\mu_{i}-2\sigma_{i},\mu_{i}+2\sigma_{i}\right]\\ V_{x,o}+1 & \text{otherwise}, \end{array} \end{array}$$

where *V*_*x*,*a*_ represents affirmative vote and *V*_*x*,*o*_ represents negative vote. If intensity of pixel is located in [*μ* − 2*σ*, *μ* + 2*σ*], the *V*_*x*,*a*_ increases by one. Otherwise, *V*_*x*,*o*_ increase by one. The total votes are equal to the number of statistical features *V*_*x*,*a*_ + *V*_*x*,*o*_ = *n*.

Based on the votes and probability density set, the actual probability density of a pixel within search area is confirmed as

(10) $$\begin{array}{c} s\left(x\right)=\{\begin{array}{cc} P_{x,\max} & V_{x,a}>V_{x,o}\\ P_{x,\min} & V_{x,a}\leq V_{x,o}, \end{array} \end{array}$$

where *P*_*x*,max⁡_ is the maximal value in probability density set and *P*_*x*,min⁡_ is the minimum value. If affirmative votes are more than negative votes, the probability density of a pixel is set to maximum in probability density set. On the contrary, it is set to minimum in probability density set.

A probability density map *s*(*I*_*m*_) is generated after probability density of all pixels within search region is ascertained using voting mechanism. It is used to limit oversegmentation. The *S*(*ϕ*) term can speed up the propagation motion of zero level set when the initial contour is far away from the desired object boundaries.

Moreover, the second energy term *L*_*g*_(*ϕ*) represents edge force which pushes the initial curve towards the boundaries of the object. It is defined as

(11) $$\begin{array}{c} L_{g}\left(\phi \right)=\int_{\Omega }g\left(I\right)\delta_{\varepsilon }\left(\phi \right)\left|\nabla \phi \right|dx, \end{array}$$

where *g*(*I*) is an edge detection function which is defined as

(12) $$\begin{array}{c} g\left(I\right)=\frac{1}{1+{\left|\nabla \left[G*I\right]\right|}^{2}}, \end{array}$$

where *G* is Gaussian filtering operator. ∗ means convolution. *I* is the CT image. Edge force is minimized when the contour of zero level set is located at boundaries of object, because edge detection function takes small value at boundaries.

In order to generate an optimal initial level set, which can satisfy the location requirement of initial zero level set, we apply a mask of previous slice to define the initial contour of zero level set. Since the shape variation is not obvious between two adjacent slices, the extracted object region of previous slice is regarded as a priori shape mark. The binary mask shrinks *k* pixel along its shape to generate an initial contour (See Figure 1(e)). The initial contour is located in the object region of current slice, because location of object organ in adjacent slices is similar.

The initial level set function (LSF) *ϕ*_0_ is defined as a binary step function:

(13) $$\begin{array}{c} \phi_{0}\left(x\right)=\{\begin{array}{cc} -c, & \text{if}\;\;x\in R_{0}\\ c, & \text{otherwise}, \end{array} \end{array}$$

where the *R*_0_ is the initial contour region. *c* is a constant set to 2.

The level set evolution equation in a priori based distance regularity level set is finally defined by

(14) ∂ϕ∂t=μdiv⁡(dp(|∇ϕ|∇ϕ)+αs(Im)δε(ϕ)+λδε(ϕ)div⁡(g(I)∇ϕ|∇ϕ|)),

where div⁡(·) is the divergence operator and *d*_*p*_ is a function defined in [11]:

(15) $$\begin{array}{c} d_{p}\left(s\right)≜\frac{p^{\prime }\left(s\right)}{s}. \end{array}$$

### 2.4. Object Organ Segmentation

A priori based distance regularity level set method is applied to extract the object organ in CT images. Since the intensity distribution of the object organ is irregular due to the noise caused in the image formation stage, a Gaussian blur filter is used to reduce the noise in preprocess. The steps of segmentation process are shown in Figure 3.

1. Initialize the training dataset by manually segmenting a slice in which object organ has a largest cross-section in input abdominal CT volume. Its next slice is the first one to segment.
2. Based on training dataset, generate the statistical feature set which is regarded as a priori knowledge and used to guide segmentation of pancreas.
3. Reduce the noise in CT slice using a Gaussian blur filter.
4. Generate a search region based on mask of previous slice and then calculate the probability density map using voting mechanism.
5. Generate an optimal initial zero level set based on mask of previous slice.
6. Based on optimal initial zero level set, extract the object using a priori based distance regularity level set method.

The extracted object will be added into training dataset as a priori knowledge to guide the segmentation of its next slice.

In practical process of object segmentation, a two-phase segmentation scheme is employed to get a better result. The first phase can be seen as a high speed level set evolution and the second phase can be seen as a high accurate level set evolution. In the first phase, the zero level set is initialized as a binary step function using function (13). The level set evolution follows function (14). After the first phase, the zero level set contour is closed to the object boundary. In the second phase, the main purpose is to accurately extract the object region. The level set evolution equation is reset as

(16) $$\begin{array}{c} \frac{\partial \phi }{\partial t}=\mu \mathrm{div}\left(d_{p}\left(\left|\nabla \phi \right|\nabla \phi \right)+\lambda \delta_{\varepsilon }\left(\phi \right)\mathrm{div}\left(g\left(I\right)\frac{\nabla \phi }{\left|\nabla \phi \right|}\right)\right). \end{array}$$

Because the energy term *S*(*ϕ*) pushes the initial contour toward the final boundary in a high speed, it is likely to make the contour across the object boundary and then cause oversegmentation. Thus, it is abolished in the second phase.

Through amount of experiment, we empirically define some values of parameters of great significance to optimize the segmentation result. In this configuration of parameters, the average similarity index of all segmentation results can get a high rate (SI = 0.922, introduced in Section 3.1).

In the first phase, *u* = 0.2, *λ* = 3, and *α* = −1 are employed in (14). A small coefficient *α* for the energy term *S*(*ϕ*) is to restrict contour expanding too rapidly and preserve the zero level set contour from crossing the boundary of object region. The iterator time in first phase is set between 5 and 10.

In the second phase, the zero level set contour is closed to the boundary of object, such that *u* = 0.2, *λ* = 2, and *α* = 0 are employed. Level set evolution is dominated by edge force. A large weight is assigned to energy term *L*_*g*_(*ϕ*), which means a stronger constraint force of boundary pushes zero level set curve towards final boundary while limiting the oversegmentation of object region. The iterator time is set between 3 and 5 in this phase.

The segmentation results of different shape and acreage of object are controlled by adjusting the iteration time. Moreover, the parameters can be fine-tuned to adapt with different CT volume to get an optimal result.

## 3. Results and Discussion 

The proposed method is compared to geodesic active contour method (GAC), geodesic active without edge method (C-V), shape a priori based level set method (SPLS), a hybrid level set method (HLS), a shape detection level set method (SDLS), confident connected region growing method (CCRG), and improved variational level set method (IVLS). Our method is referred to as PBDR. Our method, GAC method, shape detection level set method, shape a priori based level set method, and improved variational level set method are implemented using C/C++ language. C-V method and HLS method are implemented in Matlab code. All methods run on a desktop PC with 8 GB RAM and 2.4 GHz Intel Core i7 processer. The same preprocess are applied to all methods.

The trade-off between number of manual labelling and algorithm efficiency of proposed method is also evaluated. Based on a volume with 161 CT abdominal images, different numbers of manual labelling are applied as a priori knowledge to guide the segmentation.

### 3.1. Performance Measure Standard

For evaluation of efficiency and accuracy, three measures, (1) false positive error (FPE), (2) false negative error (FNE), and (3) the similarity index (SI), are used to measure the performance of methods.

False positive error [16] is defined as the ratio of the total number of extracted object region pixels outside the golden standard region to the total number of golden standard of object region:

(17) $$\begin{array}{c} \text{FPE}=\frac{N\left(O\right)\cap N\left(B\right)}{N\left(G\right)}\times 100\%, \end{array}$$

where *O* represents the pixels of extracted object region. *G* represents the golden standard of object organ. *B* represents the remaining areas except the region of golden standard in the CT image. *N*(*O*)∩*N*(*B*) represents the total number of extracted object region pixels outside the golden standard region. *N*(*G*) represents the total number of golden standard of object region.

False negative error [16] is defined as the ratio of the total number of golden standard of object outside the extracted object region to the total number of pixels of golden standard of object region:

(18) $$\begin{array}{c} \text{FNE}=\frac{N\left(G\right)-\left(N\left(O\right)\cap N\left(G\right)\right)}{N\left(G\right)}\times 100\%, \end{array}$$

where *N*(*O*)∩*N*(*G*) is total number of pixels in intersection of extracted object region and golden standard of object. *N*(*G*)−(*N*(*O*)∩*N*(*G*)) is the total number of golden standard of object outside the extracted object region.

Similarity index [17] is defined as the percentage of pixels in intersection of extracted object region and golden standard of object:

(19) $$\begin{array}{c} \text{SI}=\frac{2\left(N\left(O\right)\cap N\left(G\right)\right)}{N\left(O\right)+N\left(G\right)}\times 100\%, \end{array}$$

where *N*(*O*) is the total number of extracted object region.

### 3.2. Experimental Datasets

Three medical image datasets including pancreas dataset, liver dataset, and spleen dataset are used in evaluation. Pancreas dataset contains 10 volumes of CT image. Liver dataset contains 9 volumes of abdominal CT images. Spleen dataset contains 5 volumes of abdominal CT images. All datasets are provided by PLA General Hospital, Shenyang, China. CT images in datasets have a resolution of 515 × 512 pixels with a thickness varied between 0.6 mm and 0.7 mm. Each image in the datasets is provided corresponding golden standard manually delineated by experienced radiologists.

### 3.3. Segmentation Results and Evaluation

All the state-of-the-art medical image segmentation methods and the proposed method are applied to extract object region from the CT volume in all the medical image datasets. Average false positive error, false negative error, and similarity index are, respectively, computed for each compared method based on all segmentation results of all slices. First we calculate false positive error, false negative error, and similarity index for each segmentation results of all methods. Then average values of the three measure standard of each method are calculated based on their respective segmentation results.

**Figure 2:**
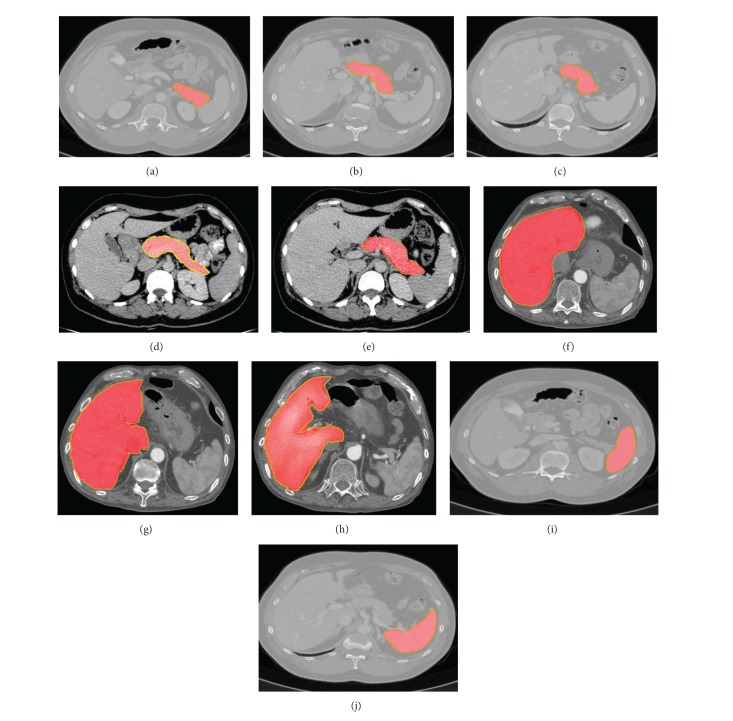
Exemplary segmentation results of our proposed method based on pancreas, liver, and spleen datasets. Red regions are segmentation results using proposed method and yellow outline marks the golden standard.

Figure 2 shows some examples of segmentation results of our method. The extracted object regions are complete and the edges are smooth.

**Figure 3:**
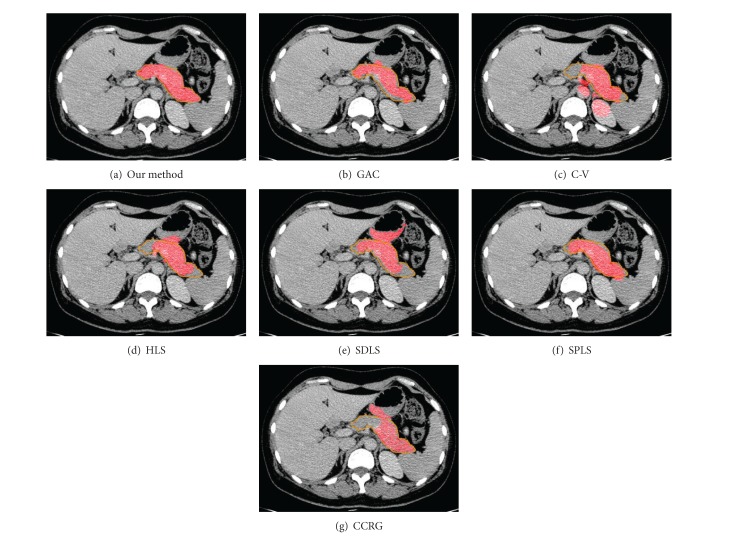
The examples of pancreas extraction result based on different methods. (a) Our method. (b) Geodesic active contour method. (c) Shape a priori based level set method. (d) Geodesic active without edge method. (e) Hybrid level set method. (f) Shape detection level set method. (f) Confident connected region growing method.

Figure 3 shows examples of pancreas extraction results based on all evaluated method.

**Figure 4:**
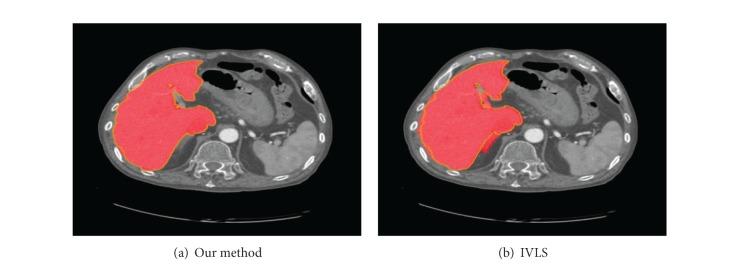
Comparison of segmentation results of our proposed method and improved variational level set method.

Figure 4 shows comparison of segmentation results of our proposed method and the improved variational level set method.

**Figure 5:**
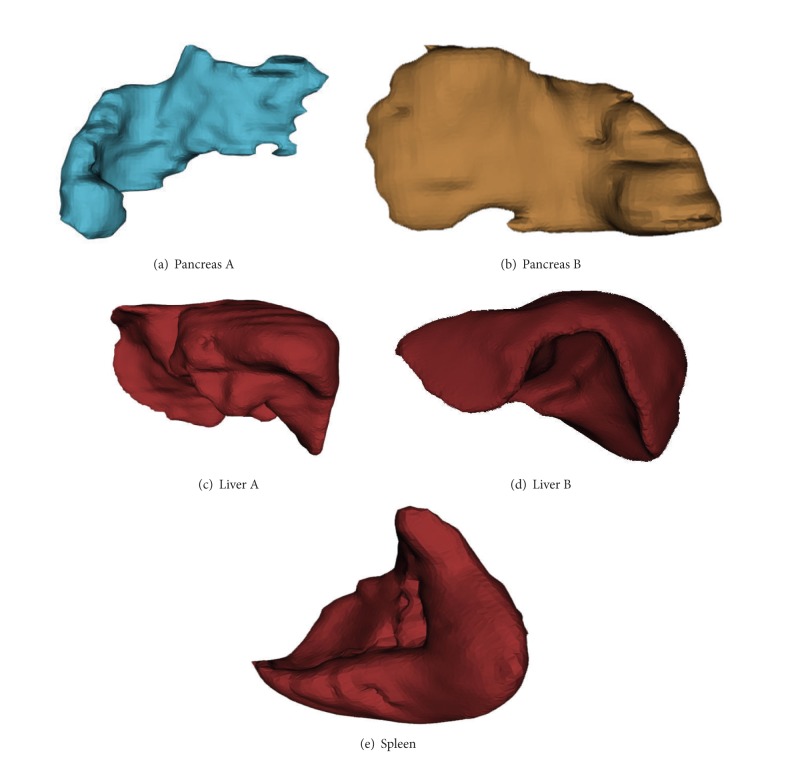
3D view of extracted organs based on our proposed method. (a), (b) 3D view of different pancreas. (c), (d) 3D view of different liver. (e) 3D view of spleen. They are reconstructed using the sequence of segmentation results based on proposed method.

Figure 5 shows 3D view of the extracted object organ using our proposed a priori based level set method.

**Figure 6:**
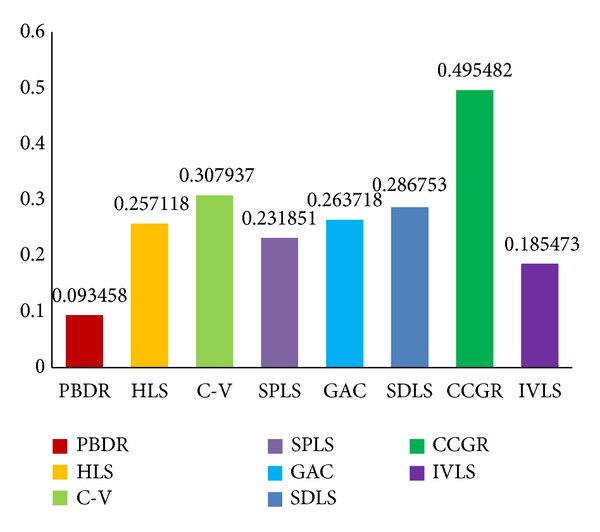
False negative error evaluation results of our method (PBDR), hybrid level set method (HLS), C-V method (C-V), shape a priori based level set method (SPLS), geodesic active contour method (GAC), shape detection level set (SDLS), confident connected region growing method (CCRG), and improved variational level set method (IVLS).

**Figure 7:**
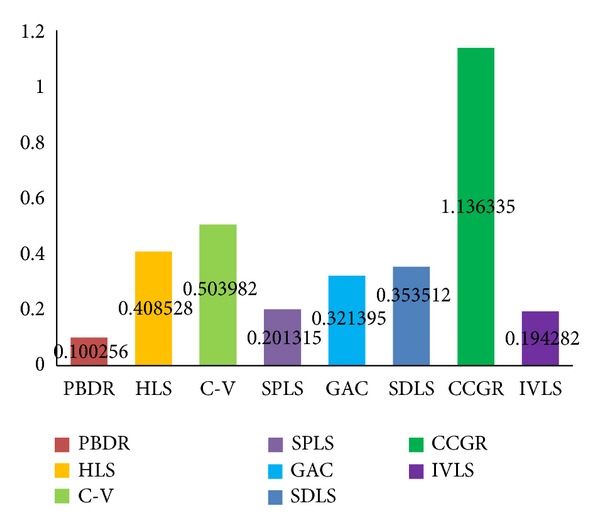
False positive error evaluation results of our method (PBDR), hybrid level set method (HLS), C-V method (C-V), shape a priori based level set method (SPLS), geodesic active contour method (GAC), shape detection level set (SDLS) method, confident connected region growing method (CCRG), and improved variational level set method (IVLS).

**Figure 8:**
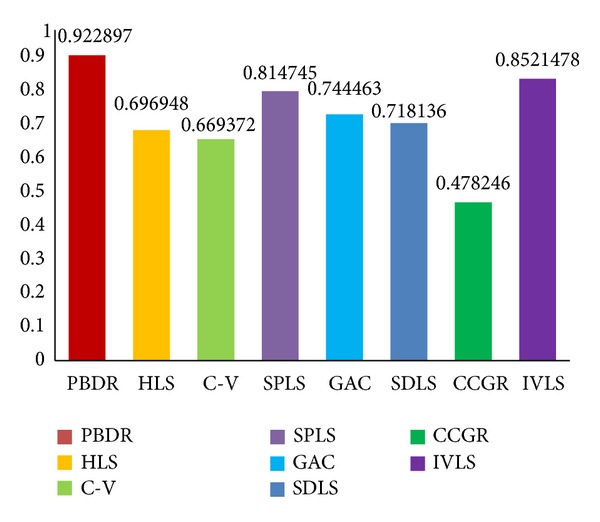
Similarity index evaluation results of our method (PBDR), hybrid level set method (HLS), C-V method (C-V), shape a priori based level set method (SPLS), geodesic active contour method (GAC), shape detection level set (SDLS) method, confident connected region growing method (CCRG), and improved variational level set method (IVLS).

Figures 6, 7, and 8 show histogram of average value of each measure standard for all compared methods.  Table 1 contains accurate value of measure standards of all the compared methods. A lower false positive error value means less pixels of background are segmented as object region, and a lower false negative error value means less golden standard of object has not been extracted. Moreover, a higher similarity index means the segmentation results are more accurate. In summary, false positive error and false negative error are lower; the segmentation result is better. Oppositely, similarity index is higher; the segmentation result is better.

Table 2 shows time efficiency of each evaluated method.  Table 3 shows trade-off between number of initial manual labelling and algorithm efficiency of proposed method.

### 3.4. Discussion

Evaluated results indicate that the proposed a priori based level set methods (FNE = 0.093, FPE = 0.100, and SI = 0.922) outperform other state-of-art methods in object organ extraction. The a priori based and edge-based level set methods are more suitable for single organ segmentation from a medical image which contains many other organs. The C-V method (FNE = 0.307, FPE = 0.503, and SI = 0.669) abandons edge constraints and intends to achieve global optimal segmentation result, but not local optimal organ. The HLS method (FNE = 0.257, FPE = 0.408, and SI = 0.696) utilizes both edge and region information to segment object. It performs better than C-V method due to the edge constraints. The GAC method (FNE = 0.263, FPE = 0.321, and SI = 0.744) and SDLS method (FNE = 0.286, FPE = 0.201, and SI = 0.718) perform better than region-based level set method, but it is easy to cause oversegmentation at week boundary.

The a priori based level set methods perform better than edge-based level set method; especially our method gets highest accuracy and makes less false segmentation. The SPLS employs a mean statistical shape model to guide the segmentation. But the mean shape cannot adapt to the huge shape variance of object organs, such that leakage problem still exists in results.

The CCRG method and IVLS method both apply statistical feature, average intensity value, and the standard deviation to guide segmentation. In CCRG method, the mean and standard deviation of intensity value are used to define a value range. Neighbor pixels whose intensity values fall inside the range are included in the object region. This rule makes the neighbor pixels whose intensity is similar with object are easily classified into object region. This causes serious oversegmentation which is difficult to control.

IVLS method uses average intensity value and the standard deviation as a constraint parameter to optimize the evolution of level set. But the statistical information is fixed and not changed through the whole segmentation process; it cannot reflect the gradual change of intensity in image sequence. This method also applied a region growing method to generate an initial object region, but the initial region is not good enough in some cases. This causes error in segmentation.

The proposed method employs a priori statistical feature set and the shape of extracted object in previous slice to guide the segmentation. A probability density map is generated based on feature set. The probability density map is used in energy term of level set evolution function to overcome problem of leakage in segmentation results. New segmented results are added into training set to update the statistical feature. A voting mechanism is used to support the update and it can reduce the effect of singular value to the statistical features. The initial contour which is product based on shape mask of previous slice can satisfy the requirement of locating initial zero level set closed to the final contour. Therefore, our a priori based distance regularization level set method outperforms other evaluated methods in object organs extraction. On the time efficiency comparison, our method is fastest and needs least time to process a slice.

In the time efficiency comparison, among all evaluated level set methods, the proposed method is the fastest (0.34 ± 0.02 sec/slice). Because the initial zero level set is closed to the final contour and probability density map makes the contour propagate of level set has a high speed. The shape detection level set method costs 0.47 ± 0.02 sec/slice and GAC method costs 0.51 ± 0.05 sec/slice. They both just need to calculate the edge feature, but not depend on region information. C-V method and HLS method need more execution time, because they depend on the global information whose calculation is time consuming.

Evaluation of trade-off between number of initial manual labelling and algorithm efficiency of proposed method indicates that equilibrium exists. Assuming that *N* big shape variations exist in a volume, the volume is divided into *N* + 1 segment. In each segment, the slice in which object organ has a largest cross section is found out and manually labelled. Such that total *N* + 1 samples are applied to guide the extraction. Under this strategy, good algorithm efficiency can be achieved while the manual labelling is marked as little as possible.

## 4. Conclusion and Future Work

The proposed method effectively incorporates a priori statistical feature of intensity distribution and a modified distance regularized level set (MDRLS) method to extract object organs from CT image. Our main contribution is coming up with a novel application strategy of a priori knowledge for segmentation and achieving better accuracy and time efficiency in object organ extraction. Our method needs fewer and simple human-computer interaction.

Based on a priori shape of previous slice, an optimal level set contour is generated for the modified distance regularized level set. A probability density map is employed in MDRLS for further preventing the oversegmentation in object region of nonideal edges. Moreover, the proposed method is simultaneously time efficient due to high speed propagation and less iteration time.

## Figures and Tables

**Table 1:** Accurate evaluation value of FPE, FNE, and SI for each method.

Method	FNE	FPE	SI
PBDR	0.093458	0.100255	0.922897
HLS	0.257118	0.408528	0.696948
C-V	0.307937	0.503982	0.669372
SPLS	0.231851	0.201315	0.814745
GAC	0.263718	0.321395	0.744463
SDLS	0.286753	0.353512	0.718136
CCRG	0.495482	1.136335	0.478246
IVLS	0.185473	0.194282	0.8521478

**Table 2:** Quantitative measure of time efficiency for each method.

Method	PBDR	HLS	C-V	SPLS
Time (sec/slice)	0.34	3.66	2.87	1.12

Method	SDLS	GAC	CCRG	IVSL

Time (sec/slice)	0.47	0.51	0.081	0.78

**Table 3:** Trade-off between number of initial manual labelling and algorithm efficiency of proposed method in one volume.

Number of labelling	SI
1	0.726
3	0.819
5	0.923
7	0.924
9	0.924
11	0.924
13	0.925
15	0.925
